# Diagnostic accuracy of serological and imaging tests used in surveillance for hepatocellular carcinoma in adults with cirrhosis: a systematic review protocol

**DOI:** 10.3310/nihropenres.13409.2

**Published:** 2024-05-02

**Authors:** Libby Sadler, Hayley Jones, Penny Whiting, Morwenna Rogers, Kelsey Watt, Matthew Cramp, Stephen Ryder, Ken Stein, Nicky Welton, Felicity Oppe, John Bell, Gabriel Rogers

**Affiliations:** 1 Population Health Sciences, University of Bristol, Bristol, England, UK; 2 Medical School, University of Exeter, Exeter, England, UK; 3 Radiology Department, Nevill Hall Hospital, Abergavenny, Wales, UK; 4 Peninsula Medical School, University of Plymouth, Plymouth, England, UK; 5 Nottingham University Hospitals NHS Trust, Nottingham, UK; 6 Patient and Public Involvement, NIHR Nottingham Biomedical Research Centre, Nottingham, UK; 7 Manchester Centre for Health Economics, The University of Manchester, Manchester, England, N13 NPL, UK

**Keywords:** Hepatocellular carcinoma, HCC, cirrhosis, AFP, ultrasound, systematic review, surveillance, protocol

## Abstract

**Background:**

Liver cirrhosis is the largest risk factor for developing hepatocellular carcinoma (HCC), and surveillance is therefore recommended among this population. Current guidance recommends surveillance with ultrasound, with or without alpha-fetoprotein (AFP). This review is part of a larger project looking at benefits, harms and costs of surveillance for HCC in people with cirrhosis. It aims to synthesise the evidence on the diagnostic accuracy of imaging or biomarker tests, alone or in combination, to identify HCC in adults with liver cirrhosis in a surveillance programme.

**Methods:**

We will identify studies through a 2021 Cochrane review with similar eligibility criteria, and a database search of MEDLINE, Embase and the Cochrane Database of Systematic Reviews. We will include diagnostic test accuracy studies with adult cirrhosis patients of any aetiology. Studies must assess at least one of the following index tests: ultrasound (US), magnetic resonance imaging (MRI), computerised tomography (CT), alpha-fetoprotein (AFP), des-gamma-carboxyprothrombin (DCP), lens culinaris agglutinin-reactive fraction of AFP (AFP-L3), a genomic biomarker, or a diagnostic prediction model incorporating at least one of the above-mentioned tests. We will assess studies for risk of bias using QUADAS-2 and QUADAS-C. We will combine data using bivariate random effects meta-analyses. For tests evaluated across varying diagnostic thresholds, we will produce pooled estimates of sensitivity and specificity across the full range of numerical thresholds, where possible. Where sufficient studies compare two or more index tests, we will perform additional analyses to compare the accuracy of different tests. Where feasible, we will stratify all meta-analyses by tumour size and patient characteristics, including cirrhosis aetiology and liver disease severity.

**Discussion:**

This review will synthesise evidence across the full range of possible surveillance tests, using advanced statistical methods to summarise accuracy across all thresholds and to compare the accuracy of different tests.

**PROSPERO registration:**

CRD42022357163

## Background

### Target condition being diagnosed

Hepatocellular carcinoma (HCC) is the most common form of liver cancer and the third most common cause of cancer deaths worldwide ^1^. Most HCCs are asymptomatic, but when present symptoms include jaundice, loss of appetite, fatigue, nausea, and abdominal pain. In England, deaths from HCC have tripled between 1997 and 2017 ^2^.

Cirrhosis of any aetiology is the largest risk factor for developing HCC, with approximately 80–90% of HCC cases having cirrhosis as a precursor condition ^3^. The most common causes of cirrhosis are infection with hepatitis B and hepatitis C, as well as lifestyle factors such as chronic alcohol consumption, diabetes and obesity ^4^. HCC develops in 1–6% of cirrhosis patients annually and is the leading cause of death among this population ^3^. As a result, periodic follow-up (‘surveillance’) for HCC is usually recommended for people with cirrhosis ^5– 8^. When diagnosed at an early stage, HCCs may be eligible for curative treatments such as transplantation or resection. This underlines the importance of early detection ^7^.

### Surveillance tests

Two main categories of tests are commonly used for the surveillance of HCC in people with cirrhosis.

1. **Imaging tests:** ultrasound (US) is safe and relatively inexpensive, and is currently recommended by the National Institute for Health and Care Excellence (NICE) for HCC surveillance. Computerised tomography (CT) and magnetic resonance imaging (MRI) scans have higher diagnostic performance but are also more expensive and time-consuming, and CT scans expose patients to harmful radiation ^9^. Although sometimes used for surveillance, they are more frequently used to confirm the diagnosis following a positive screening test.
2. **Laboratory diagnosis:** the most frequently used serum marker for HCC surveillance is alpha-fetoprotein (AFP) ^9^, which is recommended by some but not all guidance for HCC surveillance in people with cirrhosis ^5– 8^. There is a growing body of evidence for the accuracy of other markers such as lens culinaris agglutinin-reactive fraction of AFP (AFP-L3), and des-gamma-carboxyprothrombin (DCP), although these are not routinely used in clinical practice in the UK. There is also increasing interest in genomic biomarkers such as circulating microRNAs, which have developed in recent years as a novel method of early HCC diagnosis ^10^.

### Clinical pathway

Most guidance recommends abdominal US every six months ^5– 8^ for people with cirrhosis. However, there are inconsistencies in guidance on whether patients should receive AFP (some recommend both US and AFP ^8^, some recommend US ‘with or without AFP’ ^7^ and some do not recommend AFP ^5, 6^), and who should receive surveillance. For example, the American Association for the Study of Liver Diseases (AASLD) guidelines do not recommend surveillance for those with Child-Pugh class C cirrhosis due to low anticipated survival ^7^. Following surveillance testing, confirmatory imaging tests and in some cases biopsy are needed to confirm the diagnosis and severity of cancer ^7^. Biopsy is only recommended when results from diagnostic imaging are uncertain, as risks include bleeding and tumour spread ^9^.

### Rationale

This review will be conducted as part of a larger project on the cost-effectiveness of surveillance for HCC among people with cirrhosis. This systematic review is one of four work packages in this project, which also includes a systematic review to compare HCCs found under surveillance with those diagnosed incidentally or symptomatically (in terms of tumour characteristics, treatments and survival); a mathematical decision-analytic model using data from this review to estimate the lifetime costs, benefits and harms of different HCC surveillance regimens; and finally, a print-based patient decision-aid quantifying the expected benefits and harms of surveillance.

A 2021 Cochrane review evaluated the accuracy of AFP and ultrasound, alone or in combination, for diagnosing HCC in adults with chronic liver disease ^11^. However, this review was limited to just two surveillance tests and did not stratify by tumour size or patient characteristics such as cirrhosis aetiology fully e.g. they compared >80% viral aetiology with <80%. A 2022 systematic review used network meta-analysis to compare a novel multitarget HCC blood test (Mt-HBT) with US and AFP ^12^. Although this review used advanced statistical methods to compare each possible combination of tests, it is limited to just three surveillance tests, and again, did not fully stratify by important patient characteristics such as cirrhosis aetiology. Neither review estimated the accuracy of AFP across the full range of possible diagnostic thresholds.

Our research will substantially move the state of knowledge forward, by (a) identifying, appraising and synthesising evidence across the full range of possible surveillance tests using uniform standards, (b) stratifying results in consistent ways (differentiating between aetiology of underlying liver disease and size/stage of tumour detected where possible), and (c) using advanced statistical synthesis methods to summarise accuracy across all thresholds and to compare the accuracy of different tests.

### Objectives

To review the evidence on the diagnostic accuracy of imaging or biomarker tests, alone or in combination, to identify hepatocellular carcinoma (HCC) in adults with liver cirrhosis in the context of a surveillance programme.

### Secondary objectives

- To evaluate the diagnostic accuracy of biomarker tests across all possible diagnostic thresholds
- To compare the accuracy of alternative tests
- To estimate how the diagnostic accuracy of tests varies with tumour size – in particular, to estimate the accuracy of tests in detecting smaller tumours present during early stages of cancer, if sufficient data are available
- To investigate how other population characteristics (e.g. age, sex, comorbidities, different aetiologies of liver cirrhosis) affect diagnostic accuracy

## Methods

### Criteria for considering studies for this review

We will not apply date restrictions. Studies that fulfil the following criteria will be eligible for inclusion:

***Types of studies***

In the first instance, we will include both 1-gate and 2-gate diagnostic accuracy studies. 1-gate studies are cross-sectional or prospective longitudinal analyses comparing index and reference standard test results in patients with cirrhosis and unknown HCC status, whereas 2-gate studies compare index tests results in cirrhosis patients known to have HCC with index test results in cirrhosis patients without HCC ^13^. We will exclude 2-gate designs with healthy controls, as this design is likely to lead to inflated estimates of specificity ^14, 15^.

We will record the study design during the screening stage, and may exclude all 2-gate studies depending on the quantity of 1-gate studies and the overall quality of the evidence.

***Participants***

Adults (18 years or older) with cirrhosis of any aetiology who have never had HCC. We will exclude studies with healthy participants at baseline. As the review is focused on routine surveillance for HCC among people with cirrhosis (rather than testing prompted by clinical symptoms), we will exclude studies in which all participants have clinical signs and symptoms suggestive of possible HCC at baseline. For 2-gate designs, study participants with HCC must be treatment-naïve at time of testing.

***Index tests***

Imaging tests:

- Contrast enhanced ultrasound (CEUS) imaging
- Any other type of ultrasound imaging
- Multiphase HCC-specific protocol CT imaging
- MRI imaging

Conventional biomarkers:

- Any AFP test (plasma or serum samples quantified by ELISA or chemiluminescence)
- Lens culinaris agglutinin-reactive fraction of AFP (AFP-L3)
- Des-gamma-carboxyprothrombin (DCP) (AKA protein induced by vitamin K absence or antagonists II (PIVKA-II))

Genomic biomarkers:

- We will include any genomic biomarker for the detection of HCC in the first instance, provided it has been assessed in a validation cohort (rather than only a discovery cohort). If identified studies encompass a wide range of potential genomic targets addressed in single studies, we will focus our attention on those that our clinical advisers believe are most feasible to adopt in the NHS and/or those that have been studied in multiple cohorts.

We will also include validated diagnostic prediction models incorporating one or more of the index tests above. Possible examples include:

- GALAD ^16^
- Multitarget HCC blood test (mt-HBT) ^17^
- Doylestown algorithm ^18^

We will only include studies where investigators have used the prediction model as a binary classifier with a prespecified threshold; we will exclude derivation studies and those where threshold is manipulated post hoc to maximise some accuracy criterion.

***Target condition***

Hepatocellular carcinoma

***Reference standards***

We will include any reference standard in the first instance and categorise studies at the screening stage depending on the strength of their reference standard:

- Explant pathology following transplantation, where transplantation is unrelated to HCC
- Histology of resected or biopsied lesions
- Radiological follow-up (any form of imaging) to define true disease status (with at least 6 months’ follow-up following the index test)
- Radiological testing (any form of imaging) with any form of follow-up to define true disease status (with at least 6 months’ follow-up following the index test)
- Radiological testing without follow-up
- Reference standard is only performed when surveillance tests are positive
- Patients are diagnosed from medical records (no further information)
- Reference standard is unreported

We initially piloted our eligibility criteria on a small sample of studies, which highlighted the need to keep the inclusion criteria for the reference standard broad. However, if there is enough evidence, we may exclude studies with reference standards that are more likely to introduce bias. We consider the first three reference standards (explant pathology, histology and radiological follow-up) to be preferable, as they are most likely to find HCC when it is present (i.e. they are least likely to misclassify false-negative cases as true-negatives).

***Exclusion criteria***

We will exclude two-gate studies in which HCC patients have received treatment before the index test(s) was conducted, unless <5% of all included participants have received treatment, or we can isolate the data for these participants. In the first instance we will not include studies in which <100% of participants have cirrhosis, unless stratified data are presented such that it is possible to extract data relating to only participants with cirrhosis. We will consider broadening our criteria to include studies in which participants have other liver diseases, as long as a majority have cirrhosis, depending on the quantity of evidence. We will flag studies in which >50% but <100% of participants with liver disease have cirrhosis at the screening stage.

We will exclude studies in languages other than English; however, we will list those with English abstracts that appear relevant. We will not include conference abstracts in the first instance but will consider broadening our criteria if the evidence from full publications is sparse or conflicting (if necessary, with the aid of supplementary targeted searches). We will exclude studies with insufficient data to populate a 2×2 table of test results.

### Information sources

We will identify studies through two methods:

***Cochrane review***

For studies on the accuracy of AFP and ultrasound, we will use the recent Cochrane review on the same topic with similar eligibility criteria to ours to identify evidence ^11^. We will assess the 373 included studies, as well as the 292 articles excluded by the Cochrane review authors at full text, against our eligibility criteria.

***Database searches***

To identify evidence published since the Cochrane review search (June 2020) ^11^, and evidence on the accuracy of other index tests relevant to this review, we will carry out database searches.

We will search MEDLINE via Ovid, Embase via Ovid and the Cochrane Database of Systematic Reviews for relevant evidence. There will be no restrictions on language or publication period. We will only identify published evidence. Our search combines terms covering liver cirrhosis and hepatitis with terms for HCC, terms for diagnostic methods and terms for specific tests used in HCC. We will add the strategy used in the previous Cochrane search to the search up until June 2020, and remove the results. We will add back in terms for tests other than AFP or US. The search strategy as designed for MEDLINE is available as extended data ^19^.

We will check reference lists of related systematic reviews. Additionally, we will carry out forward citation-chasing of relevant past reviews using Scopus. We will run update searches no more than six months before we submit the results for publication to identify studies published while the review is being conducted.

### Selection of studies

***Cochrane review***

Two independent reviewers will assess the full texts of the 326 included studies in the Cochrane review, as well as the 292 articles excluded at full text, against our eligibility criteria using Microsoft Access ^11^. We will reach consensus through discussion or the intervention of a third reviewer.

***Database searches***

Two independent reviewers will screen titles and abstracts identified through database searching in Microsoft Access. They will select and compare potentially relevant studies, and reach consensus through discussion or the intervention of a third reviewer to determine which records we will assess at full text. Two independent reviewers will assess full-text articles to determine whether they meet the criteria for inclusion in the review. We will document articles excluded at full text along with reasons for exclusion. We will resolve disagreements between reviewers through discussion or the intervention of a third reviewer.

### Data collection and analysis

***Data extraction and management***

We will extract data using standardised data extraction forms developed in Microsoft Access 2016. We will pilot these on a small sample of papers and adapt as needed. One reviewer will carry out data extraction and a second will check extracted data for accuracy, with disagreements resolved through discussion or the intervention of a third reviewer.

We will extract the following data where reported:

1. Study characteristics (identifier, study design, sample size, study location, method of recruitment [eg surveillance programme], year(s) of recruitment)
2. Patient characteristics (age, sex, aetiology of cirrhosis, Child-Pugh score, mean size of tumour/tumour number, inclusion and exclusion criteria)
3. Index test details (test assessed, test methods/protocols, threshold(s) for test positivity/positivity criteria)
4. Reference standard details (name of test, procedure, findings)
5. 2×2 data on index test performance (number of true positives, false positives, true negatives and false negatives) at each reported threshold. If a study reports on accuracy of two or more of our index tests, we will extract full cross-classified data (e.g. 2×2×2 table for 2 index tests) where available. Otherwise, we will extract separate 2×2 tables for each index test. If count data are not available, we will construct these from reported measures of diagnostic accuracy if possible.
6. Where test result data is presented for subgroups of interest (e.g. by aetiology of cirrhosis), we will also extract these separately
7. We will extract accuracy measures e.g. sensitivity or specificity to check these are consistent with extracted cross-classified test results

***Assessment of methodological quality***

We will use the QUADAS-2 tool ^20^, and QUADAS-C for comparative accuracy studies ^21^ to assess the risk of bias and applicability of included studies as part of the data extraction process. These have domains relating to bias from participants, the index test(s), the reference standard and flow and timing. We will tailor the tools to the review with specific guidance for answering signalling questions. If at least one of the domains is rated ‘high’, we will consider the study at high risk of bias. If all domains are rated ‘low’, we will consider the study at low risk of bias. Otherwise, we will rate the study as ‘unclear’ overall. Studies will be only rated as low risk of bias for the reference standard domain if they use explant pathology following transplantation, histology of resected or biopsied lesions, or radiological follow-up with at least 6-months’ follow-up following the index test for patients without HCC. Studies will be only rated as low risk of bias for the flow and timing domain if they have ≤3 months between index test and reference standard.

***Statistical analysis and data synthesis***

For each index test, where each study reports data relating to only a single diagnostic threshold we will synthesise data using bivariate random effects meta-analysis of sensitivity and specificity ^22^, which accounts for between-study correlation between true- and false-positive results. We will assume binomial likelihoods for numbers of true positives and true negatives, avoiding problems associated with normal approximations ^23^. We will present paired forest plots of sensitivity and specificity, and plots of study-specific estimates and meta-analysis results in receiver operating characteristic (ROC) space. When diagnostic thresholds do not vary substantially across studies, we will present pooled summary estimates of sensitivity and specificity from these meta-analyses, with 95% credible ellipses representing joint uncertainty. Where there is variation in thresholds across studies, we will use the equivalence of the bivariate and hierarchical summary ROC (HSROC) models to draw summary ROC curves ^24, 25^.

For continuous biomarkers (e.g. AFP), we anticipate that many studies will report accuracy at more than one diagnostic threshold. In this situation, we will instead synthesise data using the model of Jones *et al*., which uses all available test accuracy data to produce pooled estimates of sensitivity and specificity across the full range of numerical thresholds ^26^.

Where sufficient studies are available comparing two or more index tests, we will perform bivariate meta-regression of sensitivity and specificity, with index test as a covariate, using ‘comparative’ studies only, to enable unbiased inference about how the accuracy of tests compares ^27^. Additionally, if sufficient fully cross-classified data (2×2×2 tables) are available, we will explore use of advanced meta-analysis models that synthesise these data to produce unbiased estimates of the accuracy of tests used in sequence and appropriately precise estimates of comparative accuracy ^28^. If we identify a connected network of test comparisons reporting at similar diagnostic thresholds, we will also explore use of network meta-analysis (NMA) techniques ^29, 30^.

We will take a Bayesian approach to statistical analysis, computed using WinBUGS ^31^ and/or JAGS ^32^ via R. We will use vague prior distributions across all analyses and will check for sensitivity to choice of vague priors. We will make the data and code underpinning our analyses freely available on an open-source platform (e.g. GitHub).

To illustrate the meaning of summary estimates of sensitivity and specificity, we will use these to calculate natural frequencies for hypothetical cohorts of 1,000 people with cirrhosis, at one or more indicative estimates of HCC prevalence, and positive and negative predictive values.

As standard methods for assessing publication bias are not recommended for diagnostic test accuracy reviews ^33^, we will not investigate publication bias.

***Investigations of heterogeneity and sensitivity analyses***

Where feasible, we will stratify all meta-analyses by size of tumour detected, as the accuracy of tests for detecting smaller, earlier tumours is of primary importance for surveillance. An alternative, depending on data availability, may be to stratify analyses by stage of HCC (e.g. Barcelona Clinic Liver Cancer ^34^ categories or those meeting / not meeting Milan criteria ^35^).

Where data allow, we will also use subgroup analysis and meta-regression to explore possible heterogeneity in accuracy by the following participant characteristics:

- Cause of cirrhosis
- Participant age
- Participant sex
- Liver disease severity
- Comorbidity

Where possible we will distinguish between different types of ultrasound techniques. For CT imaging, where possible we will distinguish between reporting standards e.g. LI-RADS ^36^. For MRI imaging, we will distinguish between dynamic contrast-enhanced, abbreviated and noncontrast protocols and, where possible, further distinguish between reporting standards e.g. LI-RADS ^36^. Due to considerable improvements in imaging techniques during the last several decades, we will also consider dates of studies when comparing findings.

### Dissemination

We will publish our results as part of NIHR Library’s monograph series and will also pursue stand-alone publication for this review. We will document any amendments to the protocol in appendices to these documents.

### Study status

First reviewer screening is finished and second reviewer screening is in progress. Data extraction is also underway.

## Discussion

This review aims to summarise the evidence on the diagnostic accuracy of a range of HCC surveillance tests in adults with liver cirrhosis. We will synthesise evidence across the full range of possible surveillance tests, stratify the results by important patient characteristics such as aetiology of cirrhosis and size/stage of tumour where possible, and use advanced statistical synthesis methods to summarise accuracy across the full range of thresholds and to compare different tests.

Tumour size at HCC diagnosis is one of the most influential prognostic factors affecting 5-year patient survival ^37^ as smaller tumours are more amenable to treatments such as transplantation, resection, or ablation ^7^. Both ultrasound ^38^ and AFP ^39^ have been shown to have a lower accuracy for identifying early HCC, compared with HCC at any stage. Despite this, the previously mentioned 2021 Cochrane review found that AFP could be more sensitive than ultrasound in detecting ‘resectable’ HCCs among the small number of studies that assessed this (65% [62% to 68%] vs 53% [38% to 67%]) ^11^. Some evidence also suggests that tools such as the GALAD score, which combines AFP, DCP, AFP-L3, gender and age, may be accurate in detecting early HCC ^40^. Evidence is needed to compare the full range of possible tests in a consistent way to determine the optimum method of detecting early or small HCC in a surveillance setting.

We also intend to investigate how accuracy varies by different aetiologies of cirrhosis. Current guidelines recommend ultrasound with or without AFP for HCC surveillance, independent of aetiology ^5– 8^. However, aetiology has been shown to affect the accuracy of some diagnostic tests for HCC. For example, the accuracy of AFP in patients with hepatitis may be lower than in patients with other aetiologies, as hepatitis infection can affect AFP levels ^41^. Additionally, ultrasound quality may be inferior in those with cirrhosis caused by alcohol-related liver disease (ARLD) or non-alcoholic fatty liver disease (NAFLD) ^38^. Where sufficient data are available, our review will compare the accuracy of tests in patients with different aetiologies to determine the most accurate test for HCC surveillance in each group. We anticipate there will be enough data to stratify by hepatitis B, hepatitis C, NAFLD and ARLD, but expect to find less evidence for rarer causes of cirrhosis (primary biliary cholangitis, haemochromatosis, autoimmune hepatitis, and Wilson's disease).

There are several challenges we may encounter while carrying out this review. Firstly, the evidence base is dominated by 2-gate studies, which can produce inflated estimates of test accuracy ^13^. There are additional concerns regarding the applicability of findings from 2-gate studies, as they do not recruit participants from an HCC surveillance programme, as is the case with the majority of 1-gate studies. Secondly, we are aware that there are large disparities in the quantity of evidence for different index tests. In particular, there is a vast body of evidence on the accuracy of AFP, but much less on the accuracy of imaging tests, particularly MRI and CT ^42^. 

Using an appropriate reference standard for the diagnosis of HCC is challenging. Histology is a highly accurate reference standard, but requires transplantation, which only happens in a small percentage of severe cirrhosis cases ^43^, or biopsy, which is not recommended unless necessary due to risk of bleeding ^9^. Therefore, we expect many studies to use imaging as a reference standard, which is more likely to misdiagnose patients, particularly missing early cases ^9^. Additionally, the most accurate tests are often used to confirm the HCC diagnosis following a positive surveillance test. Therefore, histological diagnoses such as biopsy, and the most accurate imaging tests such as MRI or CT, may only be used among patients already suspected of having HCC. As cirrhosis patients with no suspicion of HCC will never receive the more accurate reference standard test, we suspect some HCC cases to be missed. Consequently, we expect most studies to be at high overall risk of bias due to the difficulties with reference standards.

## Data Availability

No data are associated with this article. Figshare: MEDLINE search strategy.pdf, https://doi.org/10.6084/m9.figshare.21904866.v1 ^44^. Figshare: PRISMA-P checklist.pdf, https://doi.org/10.6084/m9.figshare.22202347.v4 ^45^. Data are available under the terms of the Creative Commons Zero "No rights reserved" data waiver (CC0 1.0 Public domain dedication).
