# Supplementary material for: Diagnostic accuracy of serological and imaging tests used in surveillance for hepatocellular carcinoma in adults with cirrhosis: a systematic review protocol
Source: NIHR Open Res. 2024 May 2;3:23. Originally published 2023 May 12. [Version 2] doi: 10.3310/nihropenres.13409.2 (PMC11320044; doi:10.3310/nihropenres.13409.2)
Supplement: Peer review history

# Reviewer report: Reviewer response for version 2

**DOI:** 10.3310/nihropenres.14733.r31662

**Published:** 2024-06-14

**Authors:** Ramesh P Arasaradnam

**Affiliations:** 1 University of Leicester, Leicester, England, UK; 2 University of Warwick, Coventry, England, UK

I believe the authors have addressed the comments satisfactorily

 Nil else to add.

Is the study design appropriate for the research question?

Yes

Is the rationale for, and objectives of, the study clearly described?

Yes

Are sufficient details of the methods provided to allow replication by others?

Partly

Are the datasets clearly presented in a useable and accessible format?

Not applicable

Reviewer Expertise:

Biomarker discovery and Implementation

I confirm that I have read this submission and believe that I have an appropriate level of expertise to confirm that it is of an acceptable scientific standard.

# Reviewer report: Reviewer response for version 1

**DOI:** 10.3310/nihropenres.14541.r30210

**Published:** 2023-09-19

**Authors:** Michael Pavlides

**Affiliations:** 1 University of Oxford, London, UK

This paper describes the protocol for a systematic review and meta-analysis for the diagnostic accuracy of index tests used as surveillance tests for HCC in people living with liver cirrhosis. This is an important area of research and as the authors point to in their paper, significant efforts have already been made to review and summarise the available evidence. The planned review described in this protocol will extend the scope of index tests that are being examined. 

 There are a couple of points that are worth considering further. Firstly, the authors want to examine whether index tests used in surveillance for HCC improve the diagnosis of HCC. However, the effectiveness of screening / surveillance programmes is better judged on whether such programmes improve mortality. The authors may therefore want to consider revising the primary objective to be to review the evidence on whether index tests used as part of surveillance for HCC in cirrhosis improve survival in these patients, with the diagnostic accuracy for HCC as a secondary objective. 

 In the discussion, the authors describe nicely the challenges they may encounter during this work, including the fact that the evidence base is dominated by 2-gate studies. It would therefore be very informative for the medical and research community if this work could also summarise what level of evidence exists to support the use of each index test. It would be very useful to know if there are any randomised controlled trials and also if there are any studies with prospective evaluation of index tests in longitudinal studies (studies where surveillance populations without HCC are followed up, index tests are measured in real time and if they are above a predefined threshold are then followed up by a reference standard test. 

 On a related point, it may be worth providing some further clarifications about the 1-gate studies that will be included. In the current protocol these are described as "cross sectional". Does this then mean that RCT and prospective longitudinal studies as the ones described above will not be included?

Is the study design appropriate for the research question?

Yes

Is the rationale for, and objectives of, the study clearly described?

Yes

Are sufficient details of the methods provided to allow replication by others?

Yes

Are the datasets clearly presented in a useable and accessible format?

Yes

Reviewer Expertise:

Hepatology, HCC, MRI, cirrhosis

I confirm that I have read this submission and believe that I have an appropriate level of expertise to confirm that it is of an acceptable scientific standard.

# Response

**Published:** 2024-03-05

**Authors:** Gabriel Rogers

**Affiliations:** The University of Manchester, Manchester, England, UK

Thank you for your careful reading of our manuscript and the points you make. We have responded to these below and updated our paper where indicated.

- This paper describes the protocol for a systematic review and meta-analysis for the diagnostic accuracy of index tests used as surveillance tests for HCC in people living with liver cirrhosis. This is an important area of research and as the authors point to in their paper, significant efforts have already been made to review and summarise the available evidence. The planned review described in this protocol will extend the scope of index tests that are being examined.
- There are a couple of points that are worth considering further. Firstly, the authors want to examine whether index tests used in surveillance for HCC improve the diagnosis of HCC. However, the effectiveness of screening / surveillance programmes is better judged on whether such programmes improve mortality. The authors may therefore want to consider revising the primary objective to be to review the evidence on whether index tests used as part of surveillance for HCC in cirrhosis improve survival in these patients, with the diagnostic accuracy for HCC as a secondary objective.

Our manuscript notes that this review is ‘part of a larger project on the cost-effectiveness of surveillance for HCC among people with cirrhosis’. As such, the outputs of the diagnostic test accuracy review will feed into other work packages that seek to quantify the long-term benefits and harms of different approaches to surveillance, including impact on life expectancy. We have added a line to our abstract to emphasise this further.

- In the discussion, the authors describe nicely the challenges they may encounter during this work, including the fact that the evidence base is dominated by 2-gate studies. It would therefore be very informative for the medical and research community if this work could also summarise what level of evidence exists to support the use of each index test. It would be very useful to know if there are any randomised controlled trials and also if there are any studies with prospective evaluation of index tests in longitudinal studies (studies where surveillance populations without HCC are followed up, index tests are measured in real time and if they are above a predefined threshold are then followed up by a reference standard test.

This will be partly addressed by a separate work package in our project, that looks at observational studies comparing HCCs found in surveillance programmes with those coming to attention in other ways. A small proportion of these are prospective in design, though most simply take cases found in different ways and compare them retrospectively.

- On a related point, it may be worth providing some further clarifications about the 1-gate studies that will be included. In the current protocol these are described as "cross sectional". Does this then mean that RCT and prospective longitudinal studies as the ones described above will not be included?

Thanks for picking this ambiguity up. We have edited our manuscript to clarify that 1-gate studies can include ‘prospective longitudinal studies’ (which would include RCTs, if they present diagnostic accuracy data – for the record, 2 meet our criteria: Kudo *et al*., Liver cancer 2019;8(4):271-80 and Pocha *et al*., APT 2013;38(3):303-12).

# Reviewer report: Reviewer response for version 1

**DOI:** 10.3310/nihropenres.14541.r29471

**Published:** 2023-09-15

**Authors:** Ramesh P Arasaradnam, Ayman Bannaga

**Affiliations:** 1 University of Leicester, Leicester, England, UK; 2 University of Warwick, Coventry, England, UK

This is an important clinical area to consider.

 Some comments to consider:

1. Pg 3 and subsequently, helpful to stick with terminology of HCC (90% of primary liver cancer) accepting that secondaries are most common.
2. Lay summary can be simplified further – it still is a difficult read (47 Flesch score).
3. Pg 3 – term liquid biopsy; this would extend beyond miRNA but even urine, or plasma for Septin-9 for example. Would these other marker also be assessed as part of this proposal?
4. Pg 4 – would be helpful to clarify which genomic markers the authors would be considering and criteria for choice.
5. Will the authors consider mortality data – AFP would appear to have some benefit in terms of prognosis and worth comparing with proposed genomic markers?
6. Would be helpful to have some definitions of proposed index and standard tests as increasingly histological diagnosis is not available and radiological diagnosis is the reference standard.
7. Remains unclear of how this proposed review adds to the detailed Cochrane review of 2021 – herein the authors propose to widen the inclusion criteria which will involve an additional 292 papers that were initially excluded in the Cochrane review. Please can the authors provide greater detail and justification for this.

Is the study design appropriate for the research question?

Yes

Is the rationale for, and objectives of, the study clearly described?

Yes

Are sufficient details of the methods provided to allow replication by others?

Partly

Are the datasets clearly presented in a useable and accessible format?

Not applicable

Reviewer Expertise:

Biomarker discovery and Implementation

We confirm that we have read this submission and believe that we have an appropriate level of expertise to confirm that it is of an acceptable scientific standard, however we have significant reservations, as outlined above.

# Response

**Published:** 2024-03-05

**Authors:** Gabriel Rogers

**Affiliations:** The University of Manchester, Manchester, England, UK

Thank you for your careful reading of our manuscript and the points you make. We have responded to these below and updated our paper where indicated.

- Pg 3 and subsequently, helpful to stick with terminology of HCC (90% of primary liver cancer) accepting that secondaries are most common.

It is only in the plain-English summary that we refer to ‘liver cancer’; we think this is an acceptable simplification, in this context. In the main text, we exclusively define the target of interest as HCC. Is that okay?

- Lay summary can be simplified further – it still is a difficult read (47 Flesch score).

Thanks for the prompt. We have done what we can, and improved the Flesch score to 67.2 (grade <7)

- Pg 3 – term liquid biopsy; this would extend beyond miRNA but even urine, or plasma for Septin-9 for example. Would these other marker also be assessed as part of this proposal?

We have removed the reference to ‘liquid biopsy’ as we can see that it introduces ambiguity. The revised text refers to ‘genomic biomarkers such as circulating microRNAs’.

- Pg 4 – would be helpful to clarify which genomic markers the authors would be considering and criteria for choice.

We state that we will consider all genomic biomarkers, in the first instance. We have clarified that we will only narrow down if we find a wide range of targets addressed in single studies. Under those circumstances, our criteria for choice are ‘those that our clinical advisers believe are most feasible to adopt in the NHS and/or those that have been studied in multiple cohorts’.

- Will the authors consider mortality data – AFP would appear to have some benefit in terms of prognosis and worth comparing with proposed genomic markers?

No; this is beyond the scope of our review, which focuses only on diagnostic accuracy. We have emphasised, elsewhere, that the review is part of a wider project that will, inter alia, seek to assess the impact of diagnosis on the full pathway, including overall survival. However, we do not have a work package that looks at prognostic value of biomarkers, in themselves.

- Would be helpful to have some definitions of proposed index and standard tests as increasingly histological diagnosis is not available and radiological diagnosis is the reference standard.

We define both index tests and reference standards under ‘Criteria for considering studies for this review’. We have clarified that our preference for explant pathology, histology, and imaging with defined follow-up as reference standards is because we consider these are most likely to find HCC when it is present.

- Remains unclear of how this proposed review adds to the detailed Cochrane review of 2021 – herein the authors propose to widen the inclusion criteria which will involve an additional 292 papers that were initially excluded in the Cochrane review. Please can the authors provide greater detail and justification for this.

We state in ‘Rationale’ that our review adds to the 2021 Cochrane review by considering a wider range of tests, and including more extensive stratification (especially accounting for tumour size) and synthesis. Our literature search retrieves many more than the 292 papers excluded by the Cochrane review (in fact, we have screened over 10,000); however, we gave special attention to these 292, as we judged that they have a high probability of being relevant to our review despite not meeting Cochrane’s eligibility criteria.
